# Mesenchymal stem cells and their conditioned medium as potential therapeutic strategies in managing comorbid anxiety in rat sepsis induced by cecal ligation and puncture

**DOI:** 10.22038/IJBMS.2022.61860.13690

**Published:** 2022-06

**Authors:** Mina Ranjbaran, Farzaneh Kianian, Mehri Kadkhodaee, Behjat Seifi, Ghorbangol Ashabi, Fariba Akhondzadeh, Maryam Adelipour, Maryam Izad, Kamal Abdolmohammadi

**Affiliations:** 1 Electrophysiology Research Center, Neuroscience Institute, Tehran University of Medical Sciences, Tehran, Iran; 2 Department of Physiology, School of Medicine, Tehran University of Medical Sciences, Tehran, Iran; 3 Department of Clinical Biochemistry, Faculty of Medicine, Ahvaz Jundishapur University of Medical Sciences, Ahvaz, Iran; 4 Department of Immunology, School of Medicine, Tehran University of Medical Sciences, Tehran, Iran; 5 MS Research Center, Neuroscience Institute, Tehran University of Medical Sciences, Tehran, Iran; 6 Department of Immunology, School of Medicine, Iranshahr University of Medical Sciences, Iranshahr, Iran

**Keywords:** Extracellular signal-regulated kinases, Inflammation, Mesenchymal stem cells, Sepsis, Sepsis-associated-encephalopathy, Serotonin

## Abstract

**Objective(s):**

Sepsis-associated encephalopathy (SAE) is a common brain dysfunction following sepsis. Due to the beneficial effects of mesenchymal stem cells (MSCs) therapy on anxiety, an extreme and early manifestation of SAE, we hypothesized that MSCs-derived conditioned medium (CM) may be able to attenuate anxiety in cecal ligation and puncture (CLP)-induced sepsis.

**Materials and Methods:**

Rats were assigned into 4 groups: sham, CLP, MSC, and CM. All animals, except in the sham group, underwent the CLP procedure to induce sepsis. Two hours after sepsis induction, the rats in MSC and CM groups, received 1×10^6^ MSCs and CM derived from the same number of cells, respectively. 48 hr after the treatments, anxiety-related behaviors were assessed, and brain and right hippocampal tissues were collected.

**Results:**

MSCs and CM enhanced the percentages of open arm entries and time spent in the open arms of the elevated plus-maze and the time spent in the light side of the light-dark box. MSCs and CM decreased the Evans blue content and decreased the IL-6 and TNF-α levels in the brain tissue samples. Reductions in the expression of 5-HT2A receptors and phosphorylation of ERK1/2 and an increase in the expression of 5-HT1A receptors in the hippocampal tissue samples were observed in the MSC and CM groups.

**Conclusion:**

MSCs and MSCs-derived CM attenuated anxiety-related behaviors to an equal extent by reducing inflammation, modifying 5-HT receptor expression changes, and inhibiting the ERK pathway. Therefore, MSCs-derived CM may be considered a promising therapy for comorbid anxiety in septic patients.

## Introduction

Sepsis is a systemic inflammatory response against infection that is demonstrated to impair the function of some vital organs (1). Sepsis-associated encephalopathy (SAE) is a multifactorial brain dysfunction that may occur after sepsis development and appears in the early stages of the disease even before any obvious damage to other organs. In spite of advancements in antibiotic therapies and critical care techniques, up to 70% of all septic patients may develop SAE (2). The symptoms of SAE vary widely from sickness behavior and delirium to coma and may further cause cognitive impairment (3). Among these symptoms, sickness behavior is the first manifestation of sepsis and affects the development, prognosis, and outcome of the disease (4). Although sickness behavior is a normal response that enables the body to cope with infectious insult, it may become deleterious when its severity or duration does not match the severity of the disease (5).

Anxiety is clinically found as a manifestation of sickness behavior in the context of sepsis that has consequences in the life quality of the patients and may later lead to social withdrawal and depression (6). Although the mechanisms underlying comorbid anxiety in sepsis are not fully elucidated, they are suggested to involve a combination of several mechanisms, mainly within the limbic system.

In the initial phase of sepsis, pro-inflammatory cytokines released in the periphery cause brain-blood barrier (BBB) alterations such as the increase of the permeability, leading to the elevated entrance of cytokines into the brain and thereby neuronal dysfunction (7). Moreover, it is known that serotonin (5-hydroxytryptamine, 5-HT) also critically contributes to the development and progression of inflammation during sepsis (8). Interestingly, research in the anxiety field has revealed that 5-HT signaling through 5-HT1A and 5-HT2A receptors, among the various 5-HT receptors, is particularly involved in mediating anxiety-related behaviors (9, 10). From a pharmacological perspective, agonists of 5-HT1A receptors and antagonists of 5-HT2A receptors exert anxiolytic effects, indicating that these receptors have opposite mechanisms of action in modulating anxiety (11). Collectively, it is hypothesized that early targeting of the mentioned mechanisms may have potential therapeutic effects on alleviating anxiety-related behaviors in septic patients.

Recently, stem cell-based therapies have drawn significant attention in experimental and clinical studies. Among stem cells used, mesenchymal stem cells (MSCs) are of great interest due to their ease of isolation and specific biological functions (12). MSCs are self-renewing and multipotent progenitors that are able to be isolated from different tissues including bone marrow and adipose tissues (13). A significant amount of evidence indicates that MSCs have various beneficial properties including anti-inflammatory and neuroprotective activities (14, 15). However, MSCs have been suggested to also exert their therapeutic effects via secreted soluble factors (16). Since a recent experimental study showed that MSCs have the capability to attenuate anxiety-related behaviors in the setting of sepsis (17), an important question arises whether MSCs-derived conditioned medium (CM) which contains soluble factors, has also the beneficial properties of MSCs in alleviating comorbid anxiety in sepsis.

Taken together, due to the early occurrence of anxiety-related behaviors in septic patients, in the present study, we studied these behaviors shortly after sepsis induction and then evaluated the anxiolytic effects of MSCs and MSCs-derived CM. Their underlying mechanisms were also evaluated.

## Materials and Methods

***Isolation and expansion of adipose tissue-derived MSCs***

MSCs were obtained from adipose tissue of the epididymis of six Wistar rats. The tissues were carefully cut into small pieces followed by incubation with 0.1% Collagenase Type I solution (Invitrogen Gibco) for 15 min at 37 ^°^C under gentle agitation. After dilution with 4 ml of culture medium (Dulbecco’s modified Eagle’s medium [DMEM] containing 15% fetal bovine serum [FBS]), the digested mixture was centrifuged at 1500 rpm for 15 min to separate the mesenchymal stem cell pellet from adipocytes. The supernatant was disposed of, and the cellular pellet was filtered by a nylon mesh (200 µm pore size) to rinse undigested tissues. Then, an aliquot of cell suspension was removed for cell culture in DMEM-High Glucose containing 15% FBS (Gibco, USA), 100 U/ml penicillin, and 100 μg/ml streptomycin in 95% humidity and 5% CO_2_ at 37 °C. About 48 hr after the start of cell culture, the first medium substitution was performed, and the non-adherent cells were removed. The medium was replaced every two or three days. When MSCs reached 80–90% confluence, they were incubated with trypsin 0.05% (Sigma, USA) and 0.02% ethylenediaminetetraacetic acid (EDTA) for a new passage and cultured for passage 2.

***MSCs characterization***

Cell surface marker expression was determined by anti-mouse monoclonal antibodies (mAb): phycoerythrin (PE)-conjugated cluster of differentiation (CD) 34; or fluorescein isothiocyanate (FITC)-conjugated CD45 and CD44; or peridinin chlorophyll protein complex (PerCP) conjugated CD90 (BioLegend, USA). For immune phenotypic analysis, passage 2 MSCs were detached by trypsin/EDTA (Sigma, USA), washed using phosphate buffer saline (PBS), and resuspended in PBS containing FBS (1%). An aliquot of suspended MSCs (100 μl) was incubated with the above-mentioned antibodies or rat immunoglobulin (Ig) G2b isotype control antibodies (BioLegend, USA) for 45 min at 4 ^°^C. After labeling the cells, their surface markers were determined by BD FACS Calibur™ flow cytometer (BD, USA) and analyzed by Flow Jo 7.6 Software.

***Osteogenic and adipogenic differentiation***

MSCs at the second passage were harvested by trypsin digestion. To evaluate the differentiation status into the osteogenic lineage, the cells were counted, seeded at 1×10^4^ cells per well on 24-well plates (SPL, Korea), and incubated at 37 ^°^C. After one day, osteogenesis differentiation medium supplemented with 100 mM dexamethasone, 10 mM β‐glycerophosphate, and 5 g/ml ascorbic acid was added to the cells. The medium was renewed every 72 hr for 21 days. After fixation in 4% paraformaldehyde, the cells were stained with Alizarin Red which identifies calcium deposits in the culture.

For adipogenic differentiation, MSCs at a density of 15×10^3^ cells per well were cultured on 24-well plates (SPL, Korea) and incubated at 37 ^°^C for one day. Then, adipogenic differentiation medium supplemented with 100 mM indomethacin, 0.5 mM 3‐isobutyl‐methylxanthine, 250 mM dexamethasone, and 5 mM bovine insulin was added to the cells. The medium was replaced every three days for 14 days. After fixation of the cells with 4% paraformaldehyde, the cells were stained with Oil Red O to evaluate the presence of adipose vacuoles.

***Preparation of MSCs-derived CM***

To prepare CM, passage 2 MSCs were incubated in serum-free culture medium, and after two days, MSCs-derived CM was harvested. Then the supernatant was centrifuged, filtered, and immediately used for intraperitoneal injection into the CM group rats. The protein concentration of MSCs-derived CM was determined by the protein assay kit (Thermo Fisher Scientific, Pierce™ BCA) to be 600-1100 μg/ml (18, 19).

***Animal study***

A total of 48 male Wistar rats (200-250 g and 6-8 weeks old) were purchased from the Department of Physiology, Tehran University of Medical Sciences. The animals were maintained in regular cages in an animal room with a constant temperature (20-22 ^°^C) and a fixed 12 hr light-dark cycle and had free access to standard lab chow and water. All processes of dealing with the animals were conducted in accordance with the Animal Ethics Committee of the Faculty of Medicine, Tehran University of Medical Sciences (Project number: 48905, Approval ID: IR.TUMS.NI.REC.1399.045).

***Procedure for sepsis induction by cecal ligation and puncture (CLP)***

The rats were intraperitoneally anesthetized by applying 100 mg/kg of ketamine and 10 mg/kg of xylazine (20, 21). The abdominal skin and muscle layer were opened to locate and exteriorize the cecum. The contents of the cecum were gently pressed against the distal portion, and the cecum was then ligated distally to the ileocecal valve and punctured twice with an 18-gauge needle to allow small drops of cecal contents to be extruded into the peritoneal cavity. Afterward, the cecum was relocated into the abdominal cavity without spreading feces onto the abdominal wall incision. After suturing the muscle layer and skin, saline was subcutaneously injected to resuscitate the rats (37 ^°^C; 3 ml/100 g body weight) followed by returning the animals to their cages. Analgesia was achieved by intramuscular administration of ketorolac (0.86 mg/kg) **(**22-24).

**Figure 1:**
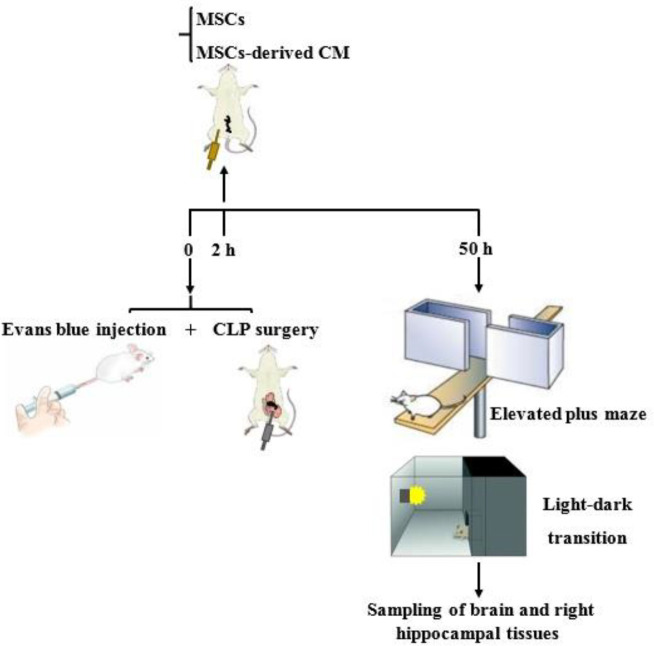
Experimental protocol of the study

The rats were randomly divided into four groups (n=12): sham, CLP, MSC, and CM. All animals except in the sham group underwent the CLP surgery for induction of sepsis. Two hours after the CLP surgery, the treatments were administered to the animals so that the MSC group rats received intraperitoneal MSCs (1×10^6^ cells, at passage 2) suspended in 50 µl saline, and the CM group rats received intraperitoneal MSCs-derived CM of 1×10^6^ MSCs. The animals in the sham group were given only an intraperitoneal injection of saline. Forty-eight hours after the treatments, the elevated plus maze and light-dark transition tests were performed. Then, the rats were anesthetized and decapitated. Brain and right hippocampal tissue samples were immediately frozen in liquid nitrogen and stored at -80 ^°^C for further analysis (Figure 1).

***Elevated plus maze test***

The elevated plus-maze test was used to evaluate anxiety-related behaviors in the rats (25). The maze included four arms (50 cm long and 10 cm wide) 50 cm above the floor: closed arms; two arms with 40-cm-high dark walls and open arms; two other arms with 1-cm-high ledges.

Two days after induction of sepsis, the animals were placed individually in the center of the maze facing one of the open arms. The entries and time spent in the open or closed arms were individually recorded for 5 min.

The percentages of open arm entries [open/total entries×100] and time spent in open arms [open / (open+closed-arm time)×100] were determined for each animal. The enhanced open arm activity (entry and time) shows reduced anxiety-related behaviors. The total entries (sum of the entries into the open and closed arms) were analyzed as a locomotor activity indicator.

***Light-dark transition test***

To examine anxiety-related behaviors, the light-dark transition test was also used (25). The box consisted of a Plexiglass box with two equal sides (30×40×40 cm), one with white walls and the other with black ones. The side with white walls was illuminated using a 60-watt bulb, while the side with black walls had a lid so it remained unilluminated. Each rat was placed in the light compartment, and the time spent there was recorded for over 5 min. The longer time spent in the light side demonstrates decreased anxiety-related behaviors.

***Evans blue dye assessment***

Permeability of the BBB was assessed by the Evans blue dye extravasation technique (26). Prior to the CLP procedure, rats (n=4) were anesthetized as mentioned above, and Evans blue dye (2% in saline, 2 ml/kg) was administered in the tail vein. Fifty hours after sepsis induction, under deep anesthesia, the thorax wall was opened, and in order to remove intravascular Evans blue, saline (200-300 ml) was transcardially infused for 20 min. Then, the animals were sacrificed, and the brain tissue samples were quickly collected, weighed, and homogenized. One milliliter supernatant liquid was blended with 2 ml trichloroacetic acid 50% (Merck, Germany) and next, centrifuged at 10000 rpm for 20 min. After diluting 1 ml of the resulting supernatant in ethanol, its optical density was measured at 620 nm with a spectrophotometer device (Biochrome, Cambridge, UK), and Evans blue concentration in the brain tissue samples was calculated as µg/g tissue.

***Inflammatory indices assessment***

Interleukin 6 (IL-6) and tumor necrosis factor-alpha (TNF-α) levels in the brain tissue samples were assessed using enzyme-linked immunosorbent assay (ELISA). All procedures were performed based on the manufacturer’s protocols (R&D Systems, Inc, USA). In these assays, the technique of quantitative sandwich enzymatic immunoassay was used. Reactions were quantified using optical density (OD) at 450/570 nm wavelength by a microplate reader (BioTek Instrument, ELX 800, Inc, USA).

***Western blot assessment***

The hippocampal tissue samples were harvested in lysis buffer, and after centrifugation at 15000 rpm for 5 min, their total protein concentration was measured according to Bradford’s method (27). Lysates, each containing 60 μg of total protein were electrophoresed by 12% SDS-PAGE and transferred onto PVDF membranes (Chemicon Millipore Co., Temecula, USA). The membranes were then blocked in 2% Electrochemiluminescence (ECL) advanced kit blocking reagent (Amersham Bioscience Co. Piscataway, USA) and incubated overnight with primary antibodies. After washing three times with Tris-buffered saline with Tween 20 (TBST) buffer, they were incubated for 1 hr at room temperature with rabbit IgG-horseradish peroxidase (HRP) conjugated secondary antibody (Cell Signaling Technology Co., New York, USA), and then the reactive bands were visualized by a chemiluminescence kit reagent (Amersham Bioscience Co., Piscataway, USA). Furthermore, the bands were analyzed using ImageJ software.

***Statistical analysis***

Data are expressed as the mean±standard error of the mean (SEM). One-way analysis of variance (ANOVA) with Tukey’s *post hoc* was used to estimate the differences among the experimental groups. *P*<0.05 was considered significant.

## Results

***Characterization and potential differentiation of MSCs***

**Figure 2:**
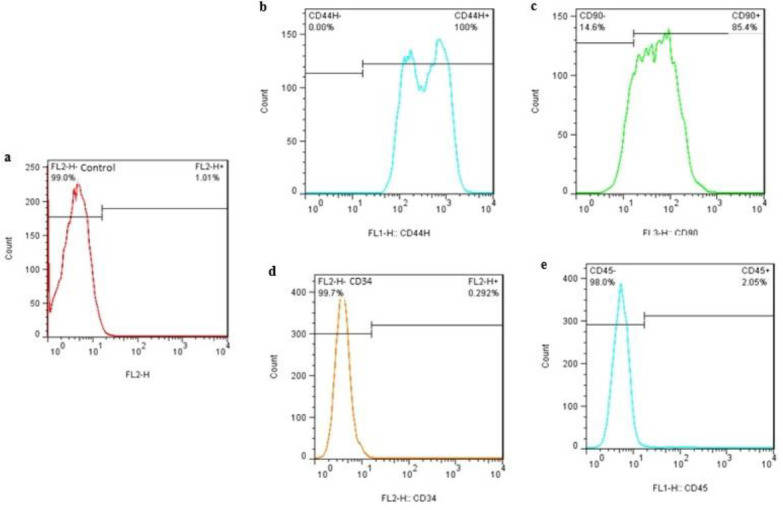
Flow cytometry analysis of cell surface markers of mesenchymal stem cells (MSCs) derived from Wistar rat adipose tissues. Non-stained MSCs (a). Passage 2 MSCs were positive for cluster of differentiation (CD) 44 and CD90 (mesenchymal markers) (b and c) while being negative for CD34 and CD45 (hematopoietic markers) (d and e)

Figure 2a shows the phenotypic analysis of non-stained MSCs. Phenotypic analysis of second passage MSCs derived from adipose tissue of the epididymis of Wistar rats by flow cytometry showed that most of those cells highly expressed CD44 and CD90 (Figures 2b and 2c, respectively), whereas having relatively lower expression of CD34 and CD45 (Figures 2d and 2e, respectively).

**Figure 3:**
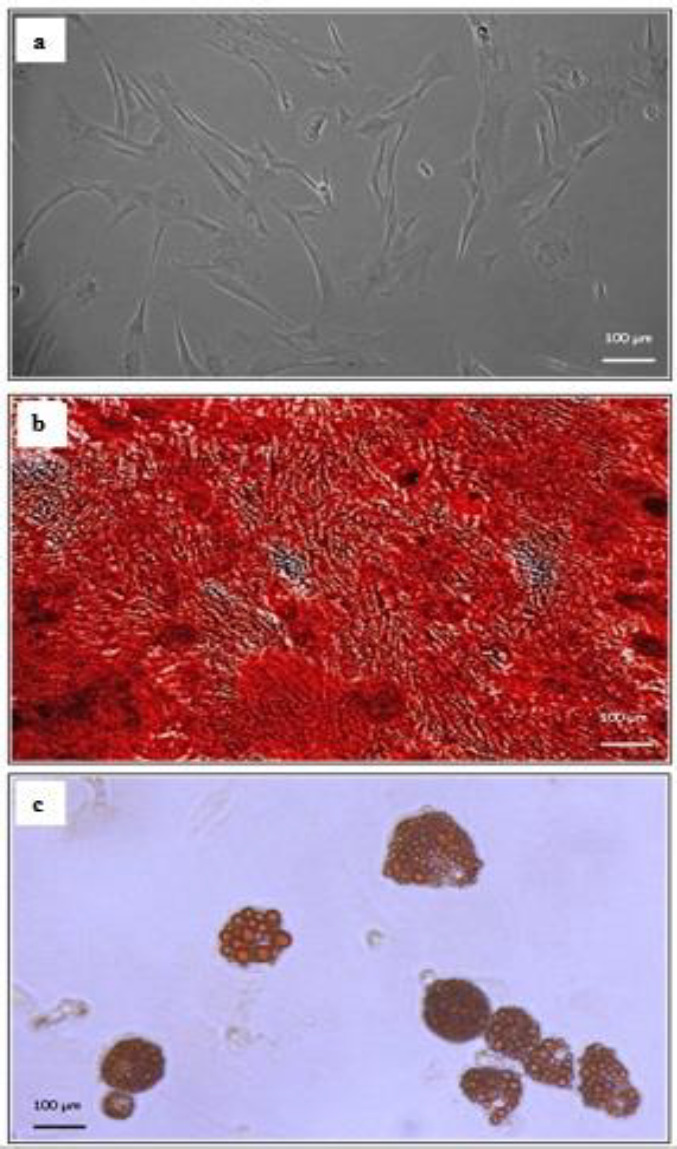
Characterization of Wistar rat adipose tissue-derived mesenchymal stem cells (MSCs). The adherent cells were mostly spindle (fibroblastic morphology) (a). Differentiation into osteocytes was evaluated by Alizarin Red staining for calcified deposits (b). Adipocyte differentiation was detected by Oil Red O staining for lipid droplets and morphological changes from spindle into round (c). Magnification of all images: × 100

Photomicrographs captured from the second passage MSCs showed that the cells mostly appeared spindle-shaped (Figure 3a). Also, photomicrographs of MSCs under osteogenic differentiation conditions revealed the formation of calcified deposits (Figure 3b). Furthermore, photomicrographs of MSCs under adipogenic differentiation conditions showed the formation of lipid droplets and morphological changes from spindle into round (Figure 3c).

***Effects of MSCs and MSCs-derived CM on open arm activity in the elevated plus-maze***

**Figure 4:**
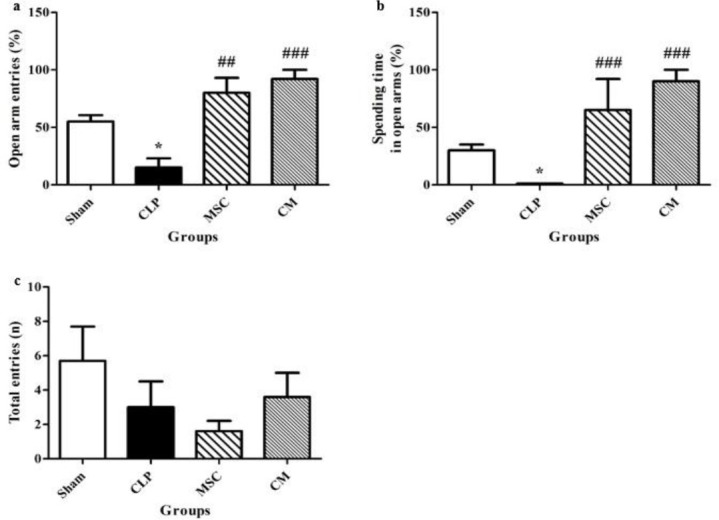
Changes of percentages of the open arm entries (a) and time spent in the open arms (b) as well as total entries (c) in the different groups. The data are expressed as mean±SEM (n= 12 in each group). * *P<*0.05 versus the sham group. ^##^*P<*0.01 and ^###^*P<*0.001 versus the CLP group

CLP-induced sepsis significantly decreased the percentage of open arm entries in comparison with the sham group (*P*<0.05, Figure 4a). MSCs and MSCs-derived CM administration significantly enhanced the percentage of open arm entries compared with the CLP group (*P*<0.01 and *P*<0.001, respectively, Figure 4a).

CLP-induced sepsis significantly decreased the percentage of time spent in the open arms compared with the sham group (*P*<0.05, Figure 4b). MSCs and MSCs-derived CM administration significantly enhanced the percentage of the time spent in the open arms compared with the CLP group (both *P*<0.001, Figure 4b). There were no considerable differences in total entries between the rats in sham, CLP, MSC, and CM groups (Figure 4c).

***Effects of MSCs and MSCs-derived CM on the time spent in the light side in the light-dark transition***

**Figure 5:**
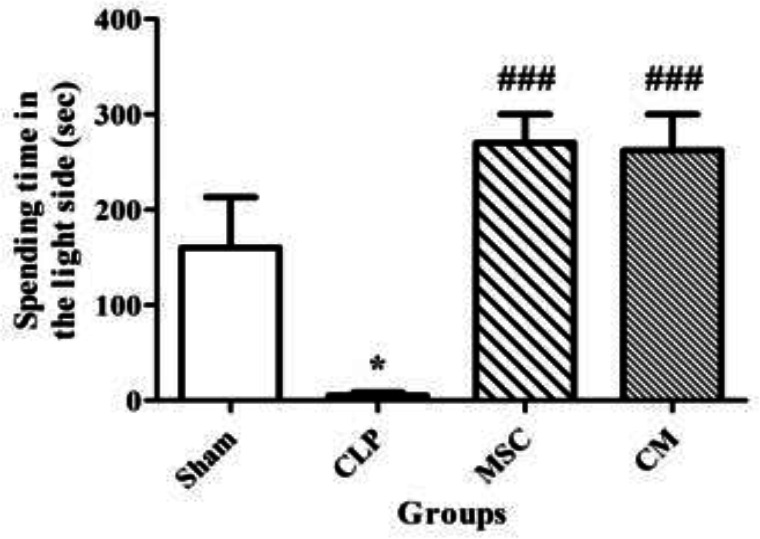
Changes in the time spent in the light side of the light-dark box in different groups. Data are expressed as mean±SEM (n=12 in each group). *P<0.05 versus the sham group. ^###^*P<*0.001 versus the CLP group

CLP-induced sepsis significantly reduced the time spent in the light side in comparison with the sham group (*P*<0.05, Figure 5). MSCs and MSCs-derived CM administration significantly increased the time spent in the light side in comparison with the CLP group (both *P*<0.001, Figure 5).

***Effects of MSCs and MSCs-derived CM on BBB permeability***

**Figure 6:**
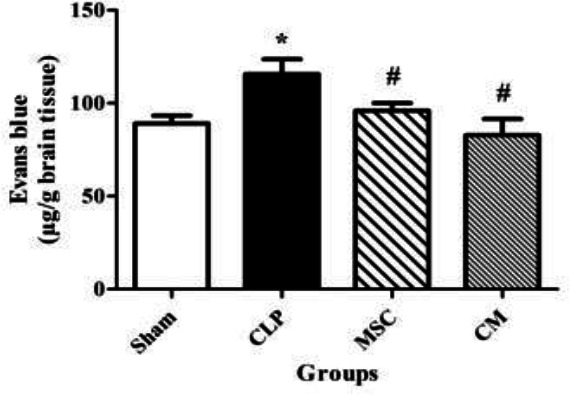
Changes of Evans blue content in the brain tissue samples in the different groups. The data are expressed as mean±SEM (n=4 in each group). *P<0.05 versus the sham group. ^#^*P<*0.05 versus the CLP group

CLP-induced sepsis significantly enhanced the Evans blue content in the brain tissue in comparison with the sham group (*P*<0.05, Figure 6). MSCs and MSCs-derived CM administration significantly decreased the Evans blue content in the brain tissue in comparison with the CLP group (both *P*<0.05, Figure 6).

***Effects of MSCs and MSCs-derived CM on inflammatory indices***

**Figure 7:**
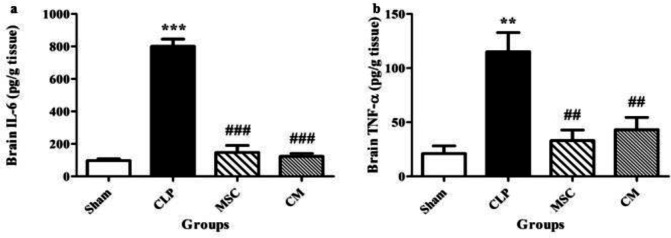
Changes in the levels of interleukin 6 (IL-6) (a) and tumor necrosis factor-alpha (TNF-α) (b) in the brain tissue samples in the different groups. The data are expressed as mean±SEM (n=6 in each group). **P<0.01 and ***P<0.001 versus the sham group. ^##^*P<*0.01 and ^###^*P<*0.001 versus the CLP group

CLP-induced sepsis significantly increased the levels of IL-6 in the brain tissue samples in comparison with the sham group (*P*<0.001, Figure 7a). MSCs and MSCs-derived CM administration significantly decreased the levels of IL-6 in the brain tissues in comparison with the CLP group (both *P*<0.001, Figure 7a).

CLP-induced sepsis significantly enhanced the TNF-α levels in the brain tissue samples in comparison with the sham group (*P*<0.01, Figure 7b). MSCs and MSCs-derived CM administration significantly reduced the increase in TNF-α levels in the brain tissues in comparison with the CLP group (both *P*<0.01, Figure 7b).

***Effects of MSCs and MSCs-derived CM on the serotonergic pathway***

**Figure 8:**
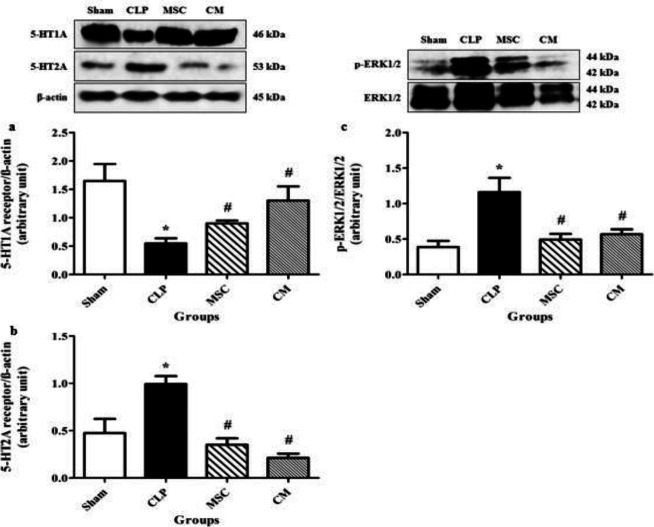
Changes in the protein expression levels of 5-HT1A receptors (a) and 5-HT2A receptors (b) as well as phosphorylation levels of extracellular signal-regulated kinases (ERK) 1/2 (c) in the hippocampal tissue samples in different groups. The data are expressed as mean±SEM (n=4 in each group). * *P<*0.05 versus the sham group. ^#^*P<*0.05 versus the CLP group

CLP-induced sepsis significantly reduced the protein expression levels of 5-HT1A receptors in the hippocampal tissues in comparison with the sham group (*P*<0.05, Figure 8a). MSCs and MSCs-derived CM administration significantly increased the protein expression levels of 5-HT1A receptors in the hippocampal tissue samples in comparison with the CLP group (both *P*<0.05, Figure 8a).

CLP-induced sepsis significantly increased the protein levels of 5-HT2A receptors in the hippocampal tissue samples in comparison with the sham group (*P*<0.05, Figure 8b). MSCs and MSCs-derived CM administration significantly decreased the protein levels of 5-HT2A receptors in the hippocampal tissue samples in comparison with the CLP group (both *P*<0.05, Figure 8b).

CLP-induced sepsis significantly increased the phosphorylation levels of extracellular signal-regulated kinases (ERK) 1/2 in the hippocampal tissue samples in comparison with the sham group (*P*<0.05, Figure 8c). MSCs and MSCs-derived CM administration significantly decreased the phosphorylation of ERK1/2 in the hippocampal tissue samples in comparison with the CLP group (both *P*<0.05, Figure 8c).

CLP: Cecal ligation and puncture, MSC: Mesenchymal stem cells, CM: Conditioned medium

CLP: Cecal ligation and puncture, MSC: Mesenchymal stem cells, CM: Conditioned medium

CLP: Cecal ligation and puncture, MSC: Mesenchymal stem cells, CM: Conditioned medium

CLP: Cecal ligation and puncture, MSC: Mesenchymal stem cells, CM: Conditioned medium

CLP: Cecal ligation and puncture, MSC: Mesenchymal stem cells, CM: Conditioned medium

CLP: Cecal ligation and puncture, MSC: Mesenchymal stem cells, CM: Conditioned medium, 5-HT: 5-Hydroxytryptamine

**Figure 9:**
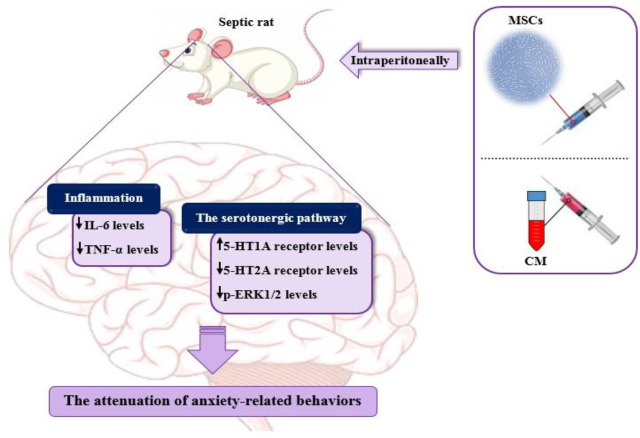
Schematic design of the beneficial effects of MSCs and MSCs-derived CM in attenuating anxiety-related behaviors following sepsis. It is possible that MSCs and MSCs-derived CM are able to decrease inflammation, modify 5-HT receptor expression changes and inhibit the ERK pathway

MSC: Mesenchymal stem cells, CM: Conditioned medium, IL-6: Interleukin 6, TNF-α: Tumor necrosis factor-alpha, 5-HT: 5-Hydroxytryptamine, p-ERK: Phosphorylated extracellular signal-regulated kinases

## Discussion

Sepsis induced by CLP is currently considered the gold standard animal model because it mimics many of the immunologic, hemodynamic, and metabolic aspects of human sepsis and thus may help to test and develop novel therapeutic agents (28). Therefore, in the current study, we applied the CLP procedure to evaluate the anxiolytic effects of MSCs and MSCs-derived CM along with their underlying mechanisms in the septic rats.

Notably, behavioral studies have indicated that anxiety is an extreme manifestation of sickness behavior in patients with sepsis (6). In our study, we observed that soon after the CLP procedure, the rats showed significant increases in anxiety-related behaviors. The elevated plus maze and light-dark transition tests were used since they are reliable tools for evaluating anxiety-related behaviors in rodents such as rats (25). The findings are consistent with the study of Jin *et al*. who indicated that induction of sepsis led to development of anxiety-related behaviors in rats (29).

In the pathophysiology of sepsis, BBB alterations associated with brain dysfunction appear in the initial phase of the disease (i.e., one day after the onset of sepsis) (7). During sepsis, infection leads to the release of a wide range of pro-inflammatory cytokines in the periphery that enter into the bloodstream and distribute throughout the body, resulting in systemic inflammation (28). This inflammation is responsible for BBB alterations such as increased permeability through destruction of the cerebral blood vessel basement membrane. The increase in BBB permeability may activate glial cells (e.g., microglia) and produce cytotoxic factors that affect the membranes, further exacerbating the damage (7). In the current study, we observed an increase of Evans blue presence in the brain tissues following sepsis, which confirmed BBB permeability enhancement. Evans blue dye is widely used to assess both basal and disease-induced increases in BBB permeability (26).

The extent of inflammation in different tissues and organs is critically involved in sepsis pathogenesis. In the central nervous system (CNS), these inflammatory reactions are known to cause vascular endothelial injury and microglial cell activation, leading to neuronal injury (30). Moreover, immune cells infiltrated during inflammation release pro-inflammatory cytokines which recruit and activate more leukocytes into inflamed sites, leading to complex immune responses and neurotoxic factor accumulation, further aggravating neuron injury (31, 32). On the other hand, some studies have interestingly reported an association between increased pro-inflammatory cytokine levels and sickness behavior (33, 34). In this line, the levels of IL-6 and TNF-α, two major pro-inflammatory cytokines, have shown to be noticeably related to the severity of anxiety (35, 36). In our study, we found elevated IL-6 and TNF-α levels in the brain tissues of rats subjected to sepsis. In agreement with our results, researchers observed an increase in brain levels of these pro-inflammatory cytokines during sepsis (37).

In the limbic system, including the hippocampus, the important role of 5-HT in emotional and behavioral processes has been known together with its implication in anxiety disorders (38). Furthermore, various receptors for 5-HT signaling are recently identified, among them 5-HT1A and 5-HT2A have drawn special attention in anxiety since alterations in the signaling of these receptors are particularly relevant to anxiety modulation (9, 10). Our study is one of the few studies that has addressed the role of 5-HT1A and 5-HT2A receptors in anxiety-related behaviors following sepsis. We found that sepsis reduced the protein expression of 5-HT1A receptors but increased 5-HT2A receptors in the hippocampus, suggesting that 5-HT2A receptors exert their anxiogenic effects by inhibiting 5-HT1A receptor expression (11). These results are in agreement with two independent studies that indicated a decrease in 5-HT1A receptors and an increase in 5-HT2A receptors in the context of anxiety-related behaviors (39, 40). In the current study, we then assessed the protein expression levels of ERK1/2 in the hippocampus of septic rats as ERK1/2 is a major downstream effector of 5-HT2A receptors (41). Previous studies have reported that 5-HT2A receptors are able to activate several downstream signaling pathways by phosphorylation of ERK1/2 (42). Importantly, the anxiogenic effects of 5-HT2A receptors are found to be exerted by triggering the ERK signaling pathways (43). In our study, sepsis led to an increase in the phosphorylation levels of ERK1/2 in the hippocampus, confirming that during sepsis, 5-HT2A receptors contribute to anxiety-related behaviors through activation of the ERK pathway by phosphorylation of ERK1/2. Similar to our findings, the study by Liu *et al*. in 2013 indicated that 5HT2A receptors affected the anxiety-related behaviors in mice subjected to LPS-induced shock by activating the ERK pathway (44).

In the current study, we aimed to compare the anxiolytic effects of MSCs and MSCs-derived CM to determine if MSCs-derived CM is as beneficial as MSCs in alleviating comorbid anxiety in sepsis. MSCs are fibroblastoid multipotent stem cells with a high capacity for differentiation and self-renewing. These cells have various biological functions such as anti-inflammatory activities, tissue repair promotion, and neuroprotection (13-15). In the initial steps, bone marrow was used as the source of these cells for use in experimental studies (45). But recently, MSCs derived from adipose tissues have become an alternative source to bone marrow because of accessibility and abundance (46). In addition, it is demonstrated that MSCs derived from adipose tissue are more suitable to treat inflammatory diseases than bone marrow-derived cells (47). Therefore, in the present study, we used adipose tissues surrounding the rat epididymis as the source of MSCs. The purity of the cells in the present study was confirmed by analysis of surface markers as there were high expressions of CD44 and CD90 (mesenchymal markers) and low expressions of CD34 and CD45 (hematopoietic markers) in MSCs derived from adipose tissue. In addition, the spindle shape of MSCs confirmed the fibroblastic morphology of the cells. The formation of Alizarin Red-positive calcified deposits, as well as formation of lipid droplets and morphological changes from spindle into round by Oil Red O staining, indicated MSCs differentiation into bone and adipocytes, respectively, demonstrating the multipotency of these cells. In agreement with Silva *et al*. observation (17), our study showed that administration of MSCs to septic rats attenuated anxiety-related behaviors, improved BBB permeability, and reduced the levels of IL-6 and TNF-α in the brain tissues. In this study, we also found that the MSCs treatment reversed down-regulation of 5-HT1A and up-regulation of 5-HT2A receptors and decreased the phosphorylation of ERK1/2 in the hippocampus of septic rats. Noteworthy, we show here, to the best of our knowledge for the first time, that MSCs-derived CM demonstrates beneficial effects similar to those of MSCs in the context of comorbid anxiety during sepsis. These effects include attenuation of anxiety-related behaviors, improvement of BBB permeability, reduction of IL-6 and TNF-α levels in the brain tissues as well as modification of the expression changes of 5-HT1A and 5-HT2A receptors and reduction of phosphorylation of hippocampal ERK1/2. The possible beneficial effects of MSCs and MSCs-derived CM, summarized in Figure 9 of the result section, and similarities in the therapeutic effects of MSCs and MSCs-derived CM are in accordance with results from a recent study (48). Thus, during sepsis, it seems reasonable to consider MSCs-derive4d CM as a more appropriate therapeutic approach to alleviating comorbid anxiety since it does not have the disadvantages associated with MSCs such as ethical concerns and the risk of immunogenicity and tumorigenicity (49).

## Conclusion

In summary, in this study on the experimental model of sepsis, the comorbid anxiety was demonstrated by increased inflammation in the brain tissues, changes in the expression of 5-HT receptors, and activation of the ERK pathway in the hippocampus. However, administration of MSCs and MSCs-derived CM, to an equal extent, returned the above parameters to the levels measured before sepsis induction. Since MSCs-derived CM has fewer disadvantages than MSCs, its administration may be more beneficial in the context of comorbid anxiety during sepsis. Nevertheless, more studies are needed to precisely examine the underlying mechanisms of beneficial effects of MSCs-derived CM.

## Authors’ Contributions

MK, BS, and MR designed the experiments; FK, GA, FA, MA, MI, and KA performed experiments and collected data; FK and MR discussed the results and strategy; MR supervised, directed, and managed the study; FK, MK, BS, GA, FA, MA, MI, KA, and MR approved the final version to be published.

## Conflicts of Interest

The authors declare that they have no conflicts of interest.

## References

[1] Angus DC, Van der Poll T (2013). Severe sepsis and septic shock. N Engl J Med.

[2] Gofton TE, Young GB (2012). Sepsis-associated encephalopathy. Nat Rev Neurol.

[3] Chung H-Y, Wickel J, Brunkhorst FM, Geis C (2020). Sepsis-associated encephalopathy: From delirium to dementia?. J Clin Med.

[4] Mazeraud A, Righy C, Bouchereau E, Benghanem S, Bozza FA, Sharshar T (2020). Septic-associated encephalopathy: a comprehensive review. Neurotherapeutics.

[5] Dantzer R (2004). Cytokine-induced sickness behaviour: A neuroimmune response to activation of innate immunity. Eur J Pharmacol.

[6] Heming N, Mazeraud A, Verdonk F, Bozza FA, Chretien F, Sharshar T (2017). Neuroanatomy of sepsis-associated encephalopathy. Critical Care.

[7] Danielski LG, Della Giustina A, Badawy M, Barichello T, Quevedo J, Dal-Pizzol F (2018). Brain barrier breakdown as a cause and consequence of neuroinflammation in sepsis. Mol Neurobiol.

[8] Du Z, Ma L, Zhen L, Sun M, Dong X, An H (20019). Research progress on the mechanism of 5-hydroxytryptamine in sepsis. Zhonghua Wei Zhong Bing Ji Jiu Yi Xue.

[9] Heisler LK, Chu H-M, Brennan TJ, Danao JA, Bajwa P, Parsons LH (1998). Elevated anxiety and antidepressant-like responses in serotonin 5-HT1A receptor mutant mice. Proc Natl Acad Sci USA.

[10] Ursano RJ, Zhang L, Li H, Johnson L, Carlton J, Fullerton CS (2009). PTSD and traumatic stress: from gene to community and bench to bedside. Brain Res.

[11] Xiang M, Jiang Y, Hu Z, Yang Y, Du X, Botchway BO (2019). Serotonin receptors 2A and 1A modulate anxiety-like behavior in post-traumatic stress disordered mice. Am J Transl Res.

[12] Gugjoo MB, Sharma GT (2019). Equine mesenchymal stem cells: properties, sources, characterization, and potential therapeutic applications. J Equine Vet Sci.

[13] Viswanathan S, Shi Y, Galipeau J, Krampera M, Leblanc K, Martin I (2019). Mesenchymal stem versus stromal cells: International Society for Cell & Gene Therapy (ISCT®) Mesenchymal Stromal Cell committee position statement on nomenclature. Cytotherapy.

[14] Wang L, Yang M, Zhang Y, Yuan R, Kang H (2019). Application of mesenchymal stem cells in sepsis. Zhonghua Wei Zhong Bing Ji Jiu Yi Xue.

[15] Zhang Y, Deng Z, Li Y, Yuan R, Yang M, Zhao Y (2020). Mesenchymal stem cells provide neuroprotection by regulating heat stroke-induced brain inflammation. Front Neurol.

[16] Chae H-K, Song W-J, Ahn J-O, Li Q, Lee B-Y, Kweon K (2017). Immunomodulatory effects of soluble factors secreted by feline adipose tissue-derived mesenchymal stem cells. Vet Immunol Immunopathol.

[17] Silva AY, Amorim EA, Barbosa-Silva MC, Lima MN, Oliveira HA, Granja MG (2020). Mesenchymal stromal cells protect the blood-brain barrier, reduce astrogliosis, and prevent cognitive and behavioral alterations in surviving septic mice. Crit Care Med.

[18] Abdolmohammadi K, Mahmoudi T, Nojehdehi S, Tayebi L, Hashemi SM, Noorbakhsh F (2020). Effect of hypoxia preconditioned adipose-derived mesenchymal stem cell conditioned medium on cerulein-induced acute pancreatitis in mice. Adv Pharm Bull.

[19] Pouya S, Heidari M, Baghaei K, Aghdaei HA, Moradi A, Namaki S (2018). Study the effects of mesenchymal stem cell conditioned medium injection in mouse model of acute colitis. Int Immunopharmacol.

[20] Kianian F, Seifi B, Kadkhodaee M, Sajedizadeh A, Ahghari P (2019). Protective effects of celecoxib on ischemia reperfusion–induced acute kidney injury: comparing between male and female rats. Iran J Basic Med Sci.

[21] Lorian K, Kadkhodaee M, Kianian F, Abdi A, Seifi B (2020). Administration of sodium hydrosulfide reduces remote organ injury by an anti-oxidant mechanism in a rat model of varicocele. Iran J Basic Med Sci.

[22] Pourmirzaei F, Ranjbaran M, Kadkhodaee M, Kianian F, Lorian K, Abdi A (2021). Sperm and testicular dysfunction during cecal ligation and puncture-induced sepsis in male rats and effects of tannic acid through reducing testicular oxidative stress and inflammation. Iran J Basic Med Sci.

[23] Akdemir FN, Tanyeli A (2019). The antioxidant effect of fraxin against acute organ damage in polymicrobial sepsis model induced by cecal ligation and puncture. Turk J Med Sci.

[24] Tanyeli A, Akdemir FN, Eraslan E, Guler MC, Sebin SO, Comakli S (2022). The possible useful effectiveness of sinapic acid sepsis-induced secondary organ damage in rats. Clin Exp Health Sci.

[25] Kianian F, Sadeghipour HR, Karimian SM, Kadkhodaee M, Seifi B (2019). Protective effects of hydrogen sulfide on anxiety in ovalbumin-induced chronic asthma. Physiology and Pharmacology.

[26] Belayev L, Busto R, Ikeda M, Rubin LL, Kajiwara A, Morgan L (1998). Protection against blood–brain barrier disruption in focal cerebral ischemia by the type IV phosphodiesterase inhibitor BBB022: a quantitative study. Brain Res.

[27] Bradford MM (1976). A rapid and sensitive method for the quantitation of microgram quantities of protein utilizing the principle of protein-dye binding. Anal Biochem.

[28] Seemann S, Zohles F, Lupp A (2017). Comprehensive comparison of three different animal models for systemic inflammation. J Biomed Sci.

[29] Jin P, Deng S, Tian M, Lenahan C, Wei P, Wang Y (2021). INT-777 prevents cognitive impairment through activating Takeda G protein-coupled receptor 5 (TGR5) by attenuating neuroinflammation via cAMP/PKA/CREB signaling axis in a rat model of sepsis. Exp Neurol.

[30] Zaghloul N, Addorisio ME, Silverman HA, Patel HL, Valdes-Ferrer SI, Ayasolla KR (2017). Forebrain cholinergic dysfunction and systemic and brain inflammation in murine sepsis survivors. Front Immunol.

[31] Gonzalez H, Elgueta D, Montoya A, Pacheco R (2014). Neuroimmune regulation of microglial activity involved in neuroinflammation and neurodegenerative diseases. J Neuroimmunol.

[32] Eyre H, Baune BT (2012). Neuroplastic changes in depression: a role for the immune system. Psychoneuroendocrinology.

[33] Calsavara AJ, Costa PA, Nobre V, Teixeira AL (2018). Factors associated with short and long term cognitive changes in patients with sepsis. Sci Rep.

[34] Vogelzangs N, De Jonge P, Smit J, Bahn S, Penninx B (2016). Cytokine production capacity in depression and anxiety. Transl Psychiatry.

[35] Skelly DT, Hennessy E, Dansereau M-A, Cunningham C (2013). A systematic analysis of the peripheral and CNS effects of systemic LPS, IL-1β, TNF-α and IL-6 challenges in C57BL/6 mice. PLoS One.

[36] Salazar A, Gonzalez-Rivera BL, Redus L, Parrott JM, OConnor JC (2012). Indoleamine 2, 3-dioxygenase mediates anhedonia and anxiety-like behaviors caused by peripheral lipopolysaccharide immune challenge. Horm Behav.

[37] Tian J, Tai Y, Shi M, Zhao C, Xu W, Ge X (2020). Atorvastatin relieves cognitive disorder after sepsis through reverting inflammatory cytokines, oxidative stress, and neuronal apoptosis in hippocampus. Cell Mol Neurobiol.

[38] Bordukalo-Niksic T, Mokrovic G, Stefulj J, Zivin M, Jernej B, Cicin-Sain L (2010). 5HT-1A receptors and anxiety-like behaviours: studies in rats with constitutionally upregulated/downregulated serotonin transporter. Behav Brain Res.

[39] McKittrick CR, Blanchard DC, Blanchard RJ, McEwen BS, Sakai RR (1995). Serotonin receptor binding in a colony model of chronic social stress. Biol Psychiatry.

[40] Adamec R, Creamer K, Bartoszyk GD, Burton P (2004). Prophylactic and therapeutic effects of acute systemic injections of EMD 281014, a selective serotonin 2A receptor antagonist on anxiety induced by predator stress in rats. Eur J Pharmacol.

[41] Oufkir T, Vaillancourt C (2011). Phosphorylation of JAK2 by serotonin 5-HT2A receptor activates both STAT3 and ERK1/2 pathways and increases growth of JEG-3 human placental choriocarcinoma cell. Placenta.

[42] Kurrasch‐Orbaugh DM, Parrish JC, Watts VJ, Nichols DE (2003). A complex signaling cascade links the serotonin2A receptor to phospholipase A2 activation: the involvement of MAP kinases. J Neurochem.

[43] Franklin JM, Carrasco GA (2013). Cannabinoid receptor agonists upregulate and enhance serotonin 2A (5‐HT2A) receptor activity via ERK1/2 signaling. Synapse.

[44] Liu C, Zhang X, Zhou JX, Wei W, Liu DH, Ke P (2013). The protective action of ketanserin against lipopolysaccharide-induced shock in mice is mediated by inhibiting inducible NO synthase expression via the MEK/ERK pathway. Free Radic Biol Med.

[45] Pati S, Gerber MH, Menge TD, Wataha KA, Zhao Y, Baumgartner JA (2011). Bone marrow derived mesenchymal stem cells inhibit inflammation and preserve vascular endothelial integrity in the lungs after hemorrhagic shock. PLoS One.

[46] Ivanova-Todorova E, Bochev I, Mourdjeva M, Dimitrov R, Bukarev D, Kyurkchiev S (2009). Adipose tissue-derived mesenchymal stem cells are more potent suppressors of dendritic cells differentiation compared to bone marrow-derived mesenchymal stem cells. Immunol Lett.

[47] Chang Cl, Leu S, Sung HC, Zhen YY, Cho CL, Chen A (2012). Impact of apoptotic adipose-derived mesenchymal stem cells on attenuating organ damage and reducing mortality in rat sepsis syndrome induced by cecal puncture and ligation. J Transl Med.

[48] Akhondzadeh F, Kadkhodaee M, Seifi B, Ashabi G, Kianian F, Abdolmohammadi K (2020). Adipose-derived mesenchymal stem cells and conditioned medium attenuate the memory retrieval impairment during sepsis in rats. Mol Neurobiol.

[49] Yousefi F, Ebtekar M, Soudi S, Soleimani M, Hashemi SM (2016). In vivo immunomodulatory effects of adipose-derived mesenchymal stem cells conditioned medium in experimental autoimmune encephalomyelitis. Immunol Lett.

